# BioMog: A Computational Framework for the De Novo Generation or Modification of Essential Biomass Components

**DOI:** 10.1371/journal.pone.0081322

**Published:** 2013-12-05

**Authors:** Christopher J. Tervo, Jennifer L. Reed

**Affiliations:** Department of Chemical and Biological Engineering, University of Wisconsin-Madison, Madison, Wisconsin, United States of America; University of Erlangen-Nuremberg, Germany

## Abstract

The success of genome-scale metabolic modeling is contingent on a model's ability to accurately predict growth and metabolic behaviors. To date, little focus has been directed towards developing systematic methods of proposing, modifying and interrogating an organism's biomass requirements that are used in constraint-based models. To address this gap, the biomass modification and generation (BioMog) framework was created and used to generate lists of biomass components *de novo*, as well as to modify predefined biomass component lists, for models of *Escherichia coli* (iJO1366) and of *Shewanella oneidensis* (iSO783) from high-throughput growth phenotype and fitness datasets. BioMog's *de novo* biomass component lists included, either implicitly or explicitly, up to seventy percent of the components included in the predefined biomass equations, and the resulting *de novo* biomass equations outperformed the predefined biomass equations at qualitatively predicting mutant growth phenotypes by up to five percent. Additionally, the BioMog procedure can quantify how many experiments support or refute a particular metabolite's essentiality to a cell, and it facilitates the determination of inconsistent experiments and inaccurate reaction and/or gene to reaction associations. To further interrogate metabolite essentiality, the BioMog framework includes an experiment generation algorithm that allows for the design of experiments to test whether a metabolite is essential. Using BioMog, we correct experimental results relating to the essentiality of *thyA* gene in *E. coli*, as well as perform knockout experiments supporting the essentiality of protoheme. With these capabilities, BioMog can be a valuable resource for analyzing growth phenotyping data and component of a model developer's toolbox.

## Background

The accuracy of constraint-based model predictions (e.g., of growth phenotypes) and the application of such models in metabolic engineering (e.g., strain design for biofuel production) are contingent on a biologically accurate biomass equation. Despite this, while numerous computational methods exist to curate genome-scale models by interrogating, hypothesizing and refining an organism's reaction, gene-reaction and/or transcriptional regulatory networks [1], [2], [3] comparatively little has been done to automate the generation and modification of an organism's biomass requirements. To fill this niche, we have developed the novel biomass modification and generation (BioMog) framework as a means to determine, *de novo*, biomass components that are consistent with large numbers (containing 1000 s of unique experiments) of high-throughput growth phenotype datasets, which are becomingly increasingly facile and inexpensive to create [4], [5].

To date, numerous methods have been created and are available to refine constraint-based models using growth phenotyping data (see [2] for recent review). SMILEY [6] works to add missing reactions to a metabolic network to correct discrepancies where the organism grows experimentally but the model does not predict growth (false negative prediction). Another approach, GrowMatch [3] groups model-data inconsistencies into false negatives (model predicted no growth, experimental growth) and false positives (model predicted growth, no experimental growth) then adds or removes metabolic reactions, respectively, to reconcile individual model-data inconsistencies. A newer approach, MIRAGE [7], makes use of metabolic flux and functional genomics data to identify reactions that may be missing from a model network. None of these methods, however, can be used to automatically test and refine a microorganism's modeled biomass (while GrowMatch [3] does include a biomass reformulation step to correct false positives it is done by manual inspection). Moreover, due to scaling issues inherent in their design, all of these methods correct single mispredictions at a time by making sequential modifications to the model without considering how these modifications will impact the overall predictive accuracy of the model. Such sequential model modifications can result in suboptimal model refinements and, thus, each proposed modification requires subsequent evaluation over the entire global dataset to ensure overall improved model performance. In addition, alternate modifications may be able to reconcile the same model-data inconsistency and the existing approaches cannot identify which of the alternate solutions would best agree with the entire experimental dataset. Thus, we have developed the BioMog (Biomass Modification and Generation) framework that can, in a scalable, parallelizable and efficient manner, consider all experimental results simultaneously to find a set(s) of biomass components that best matches experimental growth phenotypes. Consequently, BioMog proposes biomass requirements that best match all existing information while avoiding the substantial computational costs associated with existing mixed-integer linear programming (MILP) refinement approaches. Moreover, BioMog can be used to complement existing tools by suggesting other types of model adjustments to improve model predictions.

Building upon the concepts of blocked metabolites [8] (i.e., metabolites that cannot be produced or consumed by an organism), BioMog determines metabolites that are net production blocked (i.e., metabolites which cannot be generated) in a particular mutant but not in the wild type. We should note that flux through net production blocked metabolites may still occur (e.g., if the metabolite is part of a cycle) but no net production of the metabolite is possible. For simplicity, we will refer to these metabolites as blocked metabolites for the remainder of this paper. Depending on the experimental growth phenotype of the mutant in the dataset, the set of blocked metabolites can be assigned to one of two list types, assuming a correct network reconstruction:

1. Exclude Metabolite List: if growth is observed, then any blocked metabolite in the list that is included by BioMog in biomass will result in a false negative growth prediction. Metabolites within this list provide negative evidence for their inclusion in biomass.
2. Include Metabolite List: if no growth is observed, then any discovered blocked metabolites, if added to the biomass equation, will result in the model recapitulating that particular experimental result. Metabolites within this list are viable candidates for biomass components.

Once all such lists have been created, a simple integer program (IP) can be constructed to propose a new biomass *de novo* or by modifying predefined biomass equation components such that the computational agreement across all experimental observations is maximized. It is important to note that the essential biomass components proposed by BioMog are useful for qualitative predictions only (i.e., they do not take into account the relative abundance of each component and, as such, are not appropriate to use for quantitative predictions of pathway fluxes or yields without further experimental biomass composition measurements). Once this set of new biomass components has been proposed, additional experiments can be designed to interrogate the essentiality of a given metabolite using methods similar to the recently proposed FOCAL[9], which designs experiments to test the accuracy of reaction and gene-reaction relationships in constraint-based models.

Below, we describe the application of BioMog to genome-scale models for *Escherichia coli* (iJO1366) and *Shewanella oneidensis MR-1* (iSO783) using growth phenotype and fitness data for the two organisms [5], [10], [11] to propose *de novo* and modified biomass requirements. We demonstrate that the new biomass equations outperform the qualitative growth predictions of their predefined counterparts, while providing potential insights into inconsistent experiments and model structures. We subsequently designed and conducted additional experiments to interrogate the essentiality of metabolites for which the existing phenotype data provides no insight or provides conflicting results.

## Results and Discussion

**Figure 1:**
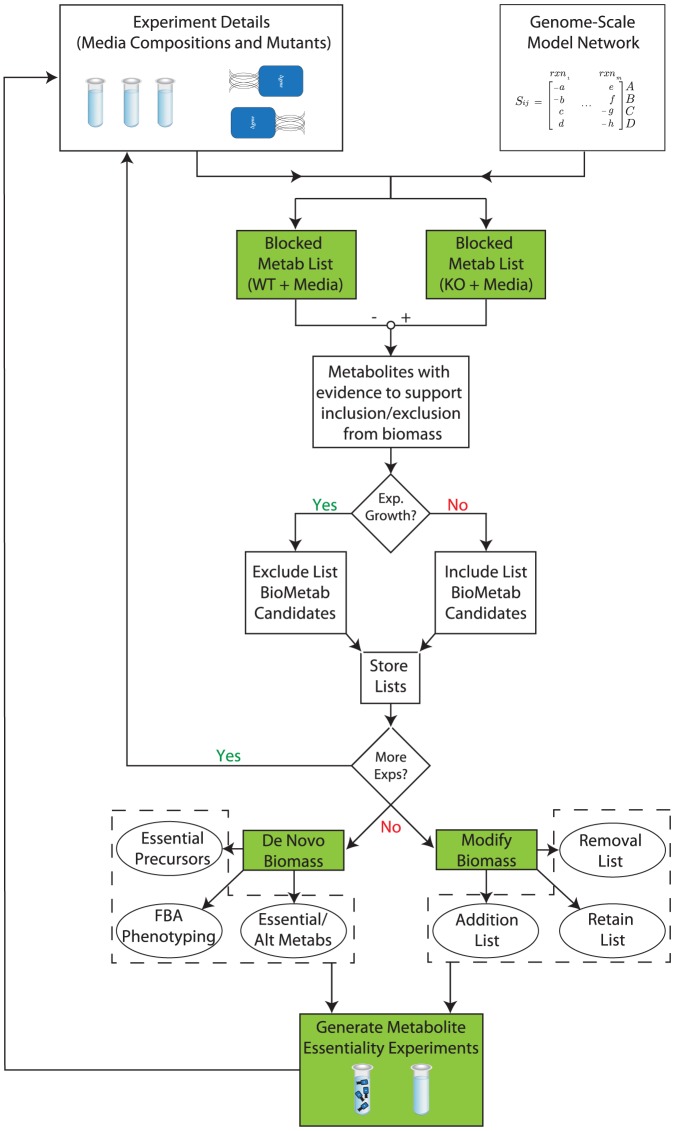
A flow diagram summarizing the BioMog framework. Initially, mutants are evaluated computationally to determine metabolites that are blocked in mutant strains but not in the wild type strain. While not strictly necessary, mutants that have substantially deleterious impacts on the network (>100 blocked metabolites) are considered uninformative and filtered out to improve the quality of proposed biomass components. Blocked metabolites are then assigned to an include/exclude metabolite list based on the experimentally observed growth phenotype. Once these lists have been created, BioMog can use this information to propose *de novo* biomass components or to modify the existing biomass equation. Additional experiments can be designed and run by BioMog to fill in any informational gaps that may exist in the current dataset after which the cycle can be repeated.

The BioMog framework uses the concept of blocked metabolites and growth phenotype data to determine potential biomass components that will yield fatal and non-fatal knockout predictions for no growth and growth experimental results, respectively. Once include and exclude metabolite lists have been generated for all experiments, an improved biomass can be computationally proposed either *de novo* or based on an existing biomass equation via the appropriate algorithm. Based on this new biomass and any relevant statistics teased from metabolite lists, BioMog can propose additional growth phenotype experiments that are designed to generate data specific to the essentiality of a particular metabolite to cellular growth. Such experiments complete the BioMog cycle and can be performed iteratively to improve both the quantity and quality of evidence supporting the essentiality of a given metabolite. This entire process is summarized in **Figure 1**.

### The BioMog Framework: An Illustrative Example

**Figure 2:**
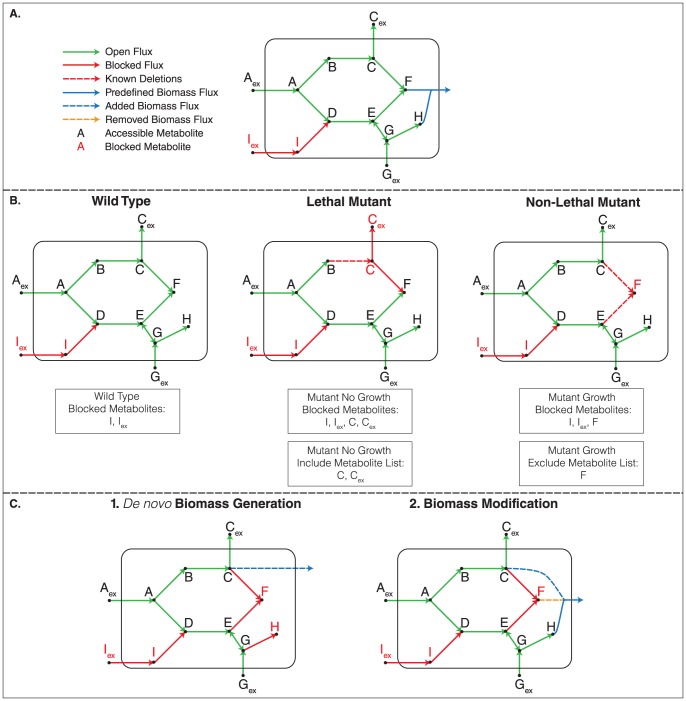
Application of BioMog to an illustrative example. (A) For an existing model and set of biomass requirements (metabolites F and H), BioMog is capable (depending on the quality and quantity of data) of generating, *de novo*, an organism's biomass requirements or of modifying a predefined biomass equation. This is accomplished by removing the initial biomass equation from the network and adding sinks for every metabolite (not shown). Blocked metabolites are determined for the wild type and mutant strains under a particular experimental condition (B). This process is repeated for all growth phenotype experiments for which data exist. The set of blocked metabolites can then be used to propose a new biomass equation or modify an existing one (C). Based on the include/exclude metabolite lists generated in this example, the original biomass equation composed of substrates F and H is modified by adding metabolite C while removing F. Since the *de novo* biomass relies solely on experimental evidence, it is important that enough data exist that test the essentiality of different metabolites if one desires an accurate and complete understanding of the biomass requirements. Here, metabolite H was absent from the proposed *de novo* biomass because no supporting or refuting evidence existed in the experimental dataset to justify its inclusion.

For illustrative purposes, we first demonstrate the BioMog process on a small reaction network (shown in **Figure 2**), where the media contains metabolites A and G (**Figure 2A**). The predefined biomass equation is removed from the network and sinks are added for every metabolite in the network. Under these conditions, the wild type network (**Figure 2B** **)** is able to produce all metabolites except for I_ex_ and I, which are blocked due to the choice of media components. I_ex_ and I should thus be removed from consideration as possible biomass components since their inclusion would suggest that the wild type would be incapable of growth. For each knockout mutant evaluated experimentally, a list of blocked metabolites (excluding those found in the wild type, i.e., I_ex_ and I) are generated and stored as include/exclude metabolite lists based on the knockout phenotype (i.e., lethal or non-lethal knockout). Once all include/exclude metabolite lists have been completed, BioMog can propose a *de novo* biomass or modify the predefined biomass using a simple integer program (IP) such that the agreement between model growth predictions and growth phenotype data is maximized (**Figure 2C**). Depending on the network structure, this process may result in new consumption blocked metabolites (i.e., a metabolite for which no consuming reaction exists) due to the removal of metabolites from the predefined biomass, which naturally behaves as an outlet for many essential metabolites.

### Application of BioMog to *Escherichia coli* and *Shewanella oneidensis* Metabolism

In order to demonstrate the efficacy and utility of the BioMog framework, the BioMog framework was applied to models of *Escherichia coli* (with ∼4200 growth phenotype experiments) and *Shewanella oneidensis* (with ∼1500 fitness experiments) metabolism, resulting in proposed biomass components that were generated *de novo* or by modifying preexisting biomass equations. BioMog's *de novo* proposed biomass components are summarized in **Table 1** while complete details of the predefined biomass modifications are provided in the supplementary materials (**Tables S1 and S2 in File S1**). In proposing these biomasses, metabolites most commonly associated with biomass such as amino and nucleic acids, as well as various currency metabolites, were weighted such that they would be selected preferentially when alternative metabolites were available (see methods for details).

**Table 1:** BioMog proposed *de novo* biomass equations for *E. coli* and *S. oneidensis*.

*E. coli* Biomass		*S. oneidensis* Biomass	
Unique	Contains Alternatives	Unique	Contains Alternatives
2,3-dihydroxybenzoylserine; 5-Formyltetrahydrofolate; L-Glutamate 5-phosphate; L-Histidine; L-Isoleucine; KDO(2)-lipid IV(A); protoheme; L-Proline	2omhmbl; [2Fe-1S] desulfurated iron-sulfur cluster; 4c2me; L-Arginine; adenosine thiamine triphosphate; Biotin; cardiolipin (tetraoctadec-11-enoyl, n-C18:1); Coenzyme A; Flavin adenine dinucleotide oxidized; L-Leucine; L-Lysine; Murein5p3p_p; nadp; pe181; L-Phenylalanine; O-Phospho-L-serine; Pyridoxamine; Siroheme; Selenite; L-Tryptophan; uagmda	L-Arginine; gdptp; L-Histidine; O-Phospho-L-homoserine	Reduced glutathione; DNA; L-Leucine; L-Methionine; 2-Oxo-3-hydroxy-4-phosphobutanoate; L-Phenylalanine; L-Proline; O-Phospho-L-serine; L-Tryptophan; L-Tyrosine

Unique metabolites were those compounds for which there was no alternative that would have maximized the objective function. Conversely, metabolites which possessed alternatives could be replaced with another compound without adversely impacting the objective function. Abbreviations used were as follows: *2omhmbl*, 2-octaprenyl-3-methyl-5-hydroxy-6-methoxy-1,4-benzoquinol, *4c2me*, 4-(cytidine 5′-diphospho)-2-C-methyl-D-erythritol, *murein5p3p_p*, two linked disacharide pentapeptide and tripeptide murein units (uncrosslinked, middle of chain), *nadp*, nicotinamide adenine dinucleotide phosphate, *pe181*, phosphatidylethanolamine (dioctadec-11-enoyl, n-C18:1), *uagmda*, undecaprenyl-diphospho-N-acetylmuramoyl-L-alanyl-D-glutamyl-meso-2,6-diaminopimeloyl-D-alanyl-D-alanine, *gdptp*, guanosine 3′-diphosphate 5′-triphosphate.

**Figure 3:**
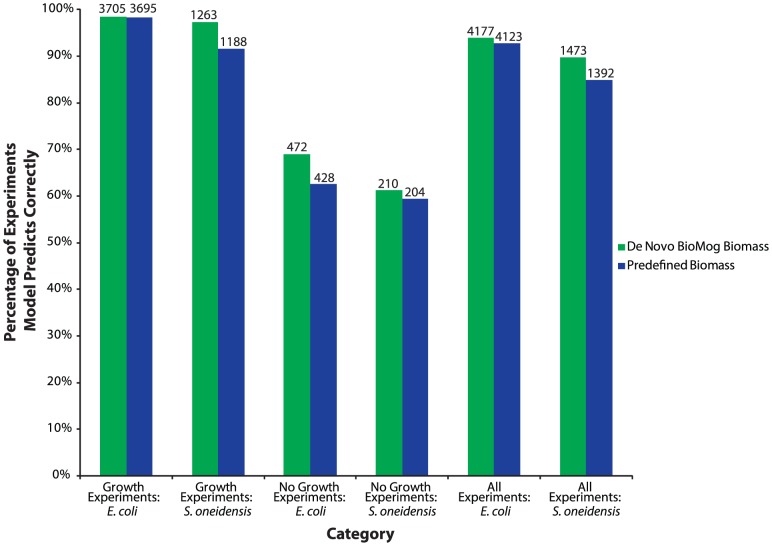
Percent of growth phenotype agreements for the BioMog De Novo biomass and the predefined biomass. Unsurprisingly, the BioMog biomass equation consistently outperforms the curated biomass over the entire dataset. Numbers reported above a given bar indicate the total number of experiments for that category.

The proposed *de novo* biomass requirements consistently outperformed their predefined counterparts at predicting the qualitative outcomes of growth phenotype experiments (**Figure 3**). Despite improvements in growth phenotype predictions, it can still be seen that there is room for improvement, particularly for mutants that did not grow (NG) experimentally. Of the 139 *E. coli* NG mutants that the *de novo* or predefined biomass equations failed to properly predict under at least one media condition, 123 were improperly predicted by both biomasses, 15 were incorrectly predicted solely by the predefined biomass and just one (*Δb1098* – a mutant defective in deoxythymidine triphosphate, dttp, biosynthesis) was incorrectly predicted only by BioMog's proposed biomass. For the 123 commonly mispredicted mutants, just under 90% did not have any blocked metabolites beyond those observed in the wild type network (implying the mutant can only result in a growth phenotype given the existing metabolic network). These mispredicted mutants could be due to experimental errors, incomplete or erroneous GPR associations, enzyme redundancy due to isozymes, metabolic redundancy due to the presence of alternate metabolic pathways which may not be used experimentally, or incomplete pathways that can be filled using methods like GapFill [8]. For example, *folA* (encoding dihydrofolate reductase) in *E. coli* has been repeatedly reported as an essential gene [10], [12], [13]; however, the present GPR association for this reaction suggests that an isozyme, *folM*, can compensate for the loss of *folA* resulting in the model having no new blocked metabolites and an erroneous growth phenotype prediction. Another experimentally essential gene, *guaB* (encoding IMP dehydrogenase) converts imp (inosine 5-phosphate) into xmp (xanthoine 5-phosphate) in guanine biosynthesis; however, the model has another route to make xmp from imp (imp → inosine → hypoxanthine → xanthine → xmp) so there are no new blocked metabolites in the 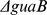
mutant over the wildtype. While structural issues in the model (e.g., GPR associations and alternative pathways) may cause some discrepancies between model predictions and data, such discrepancies can also be due to problems with the experimental data itself. For example, *sucC*, (encoding succinyl-CoA synthetase)was classified experimentally as essential; however, other reports indicate that it is non-essential [12], [13] in agreement with the model predictions. The remaining 10% of the mispredicted NG mutants did have blocked metabolites, but the inclusion of these blocked metabolites into biomass would have contradicted results of other experiments. For example, a number of mutants (*Δb0722* [*sdhD*], *Δb0588* [*fepC*], *Δb0590* [*fepD*], *Δb0592* [*fepB*]**,** *Δb1252* [*tonB*] *and Δb3919* [*tpiA*]) had Fe-enterobactin (feenter) as a blocked metabolite; however, this was excluded from BioMog's proposed biomass requirements due to numerous conflicting experimental results regarding the metabolite's essentiality (e.g., *Δb0584* [fepA], is a viable mutant where feenter is the only blocked metabolite). Consequently, to improve the qualitative predictions for these experiments it would require including additional regulatory information, repeating some conflicting experiments, or modifying the reaction network or gene-protein-reaction (GPR) associations.

**Figure 4:**
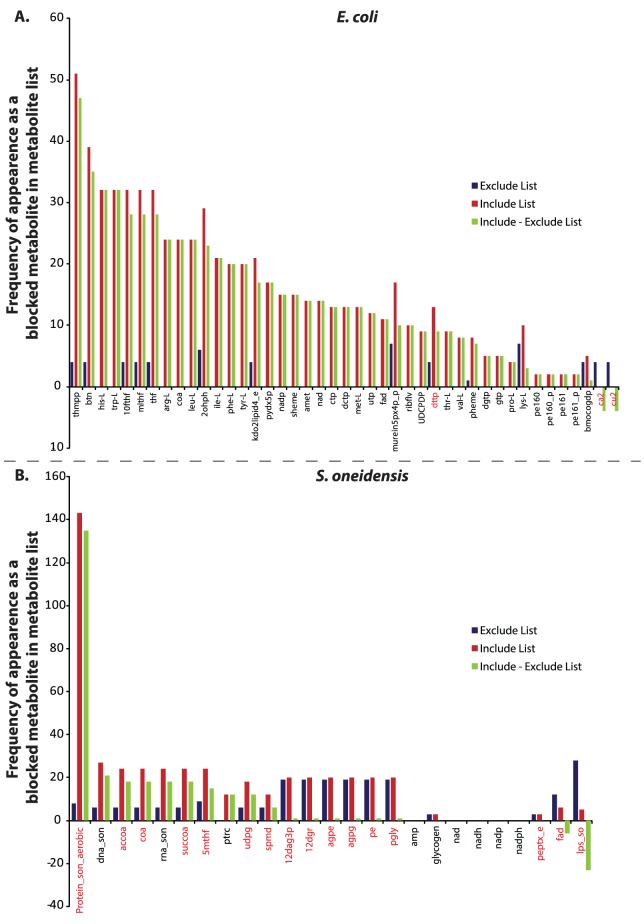
Frequency of appearance in the include/exclude metabolite lists. Frequency of select predefined biomass metabolites in include (red bars, corresponding to experiments where mutants do not grow) and exclude (blue bars, corresponding to experiments where mutants grow) metabolite lists, as well as the difference between the two (green bars) for both *E. coli* (A) and *S. oneidensis* (B). For *E. coli*, 29 of the 72 predefined components (40%) had no direct supporting or refuting experimental evidence (i.e., the red and blue bars were zero). A positive difference between the include and exclude frequency indicates a potential improvement in agreement by including the metabolite as a biomass component. Note that a positive difference does not ensure that a metabolite's inclusion will lead to a maximal agreement score. Selection of biomass components from multiple blocked metabolites associated with a given experiment can potentially replicate the same experimental phenotypes while minimizing overall disagreements (see text for details). Red labeled metabolites indicate those that were recommended for removal by the biomass modification algorithm. Metabolite abbreviations match those used in the two metabolic models.

The BioMog framework also provides information about how many experiments support/refute the essentiality of a given metabolite. This can be extracted from the frequency of appearance of a given metabolite in the include/exclude metabolite lists. These metabolite list frequencies for select predefined biomass components (*E. coli* metabolites with no experimental evidence for or against were excluded to improve visualization) are provided in **Figure 4**. Unsurprisingly, items with more experimental evidence indicating that a metabolite becomes blocked in a lethal knockout (i.e., those with high frequency in include lists) are more likely to be added to the biomass equation. Frequencies alone, however, provide an incomplete picture of selection as is evidenced by numerous modifications made to the predefined biomass equation of *S. oneidensis* (metabolites listed in red, **Figure 4B**). To completely understand BioMog decisions, it is necessary to know not only a metabolite's frequency in include/exclude lists but also the frequencies of its brethren (i.e., the other blocked metabolites that co-appear in the same include/exclude lists). Assuming a correct metabolic network, if a blocked metabolite appears in an include list with other metabolites, it is possible that the selection of one of these other metabolites may be preferable in order to obtain the most accurate biomass equation with the fewest number of components. The metabolite, protein_son_aerobic (representing an average protein molecule in the cell) in iSO783 is an example where replacing the metabolite in the predefined biomass with other brethren metabolites in the biomass improves agreement with experimental observations. In the viable mutant ΔSO2085, protein_son_aerobic is the sole blocked metabolite (beyond those observed in the wild type network) providing evidence for its exclusion from the biomass. Moreover, in a majority of no growth mutants, protein_son_aerobic is found to be blocked along with various amino acids that are precursors to protein production. As a consequence, BioMog determines that composing the biomass using essential amino acids rather than protein_son_aerobic will improve the model's qualitative growth predictions.

**Figure 5:**
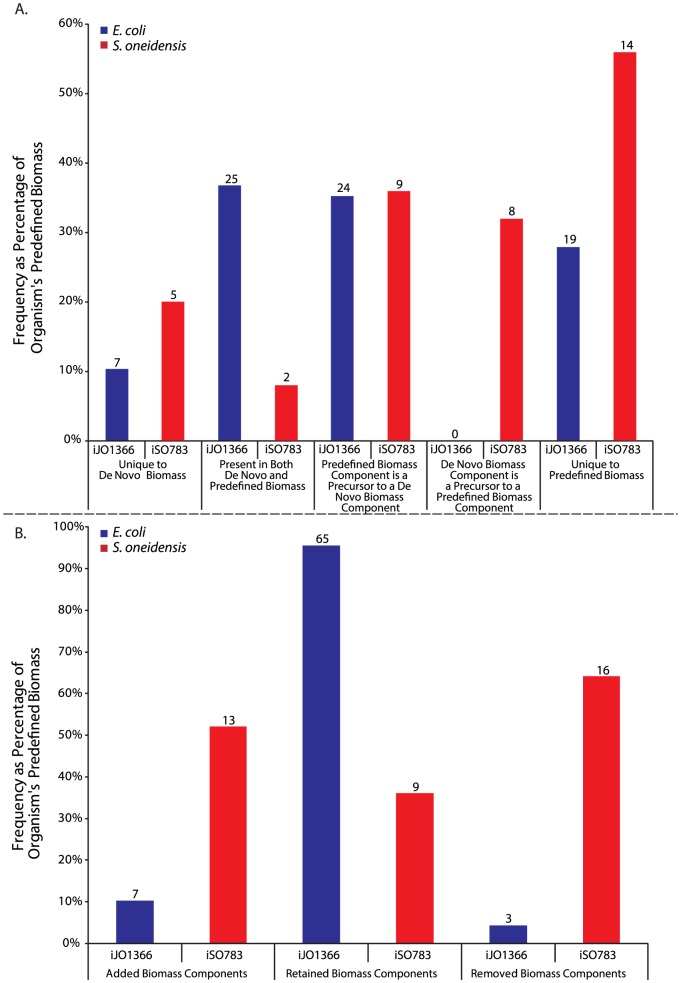
Comparison of predefined biomass to BioMog's *de novo* (top) and modified biomass (bottom). Numbers above bars indicate the number of metabolites in each category. iJO1366 and iSO783 are the genome-scale models used for *E. coli* and *S. oneidensis*, respectively.

Having evaluated the decision making process for proposing *de novo* and modified biomasses, we compared our *de novo* BioMog biomass components to the predefined model biomass components (**Figure 5**). A more detailed biomass component comparison is provided in **Tables S3 and S4 in File S1**. As can be seen for *E. coli*, over 70% of the predefined biomass components are captured in BioMog's proposed biomass, when alternative and downstream metabolites are considered. When proposing *E. coli* and *S. oneidensis* biomass requirements, BioMog would sometimes select metabolites that are related to those in the predefined biomass but are located farther downstream. While upstream predefined metabolites are not explicitly included within the BioMog proposals, they are implicitly essential under the media conditions examined when the downstream metabolites are included in the *de novo* biomass. For the *E. coli* analysis, none of the BioMog biomass components were found to be upstream of the predefined biomass components. For *S. oneidensis*, most *de novo* Biomog biomass components were downstream of predefined components or were unique to the *de novo* biomass, with the exception of eight amino acids that were included in the biomass and that were all precursors for protein_son_aerobic. For *E. coli* and *S. oneidensis*, seven and five proposed metabolites respectively were unique to the *de novo* biomass (i.e., they did not have alternatives that were either directly included or were upstream or downstream of metabolites in the predefined biomass). The unique elements of the BioMog biomasses (seven components for *E. coli* and thirteen for *S. oneidensis*, including the eight amino acid precursors) are critical to improving growth phenotype predictions and are found in both the *de novo* and biomass modification predictions (**Figure 5B**). It is important to note that the metabolites unique to the predefined biomass may be absent from the *de novo* biomass because there is no data to support their inclusion or the data suggests that these metabolites are not essential for growth.

### Inclusion of Analytical Biomass Measurements in BioMog

**Figure 6:**
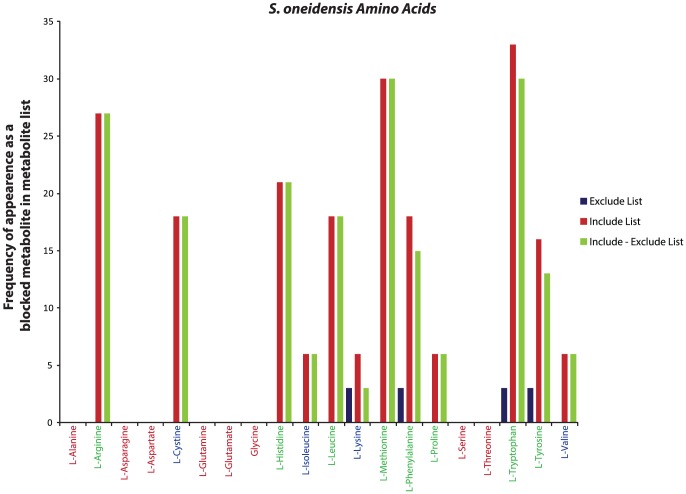
Frequency of Amino Acid appearance in the include/exclude metabolite lists for *S. oneidensis*. While twelve amino acids were not included in the original de novo biomass proposals of BioMog (Table 1), the growth phenotypes do not preclude their addition as shown here. Their exclusion by BioMog is a result of insufficient evidence for their inclusion based soley on the growth phenotype experiments performed. Metabolites in green are those that were selected by BioMog as essential biomass components. Metabolites in blue are those that have evidence to support their inclusion but were omitted because they are redundant with other metabolites for matching experimental results (e.g., isoleucine, leucine and valine biosynthesis pathways share many of the same enzymes). Metabolites colored red have no growth phenotype evidence to support their inclusion or exclusion from the biomass.

While BioMog is designed to make proposals based on growth phenotype data, it can also take into account additional experimental data such as analytically measured biomass compositions. Such data is especially useful for metabolites for which creating an informative mutant experiment would be prohibitive due to both time and complexity (see **Experimental Design** section below). This biomass composition data can be used to improve BioMog proposals, such as those for amino acids in *S. oneidensis*. It is well known that organisms generally require all twenty amino acids for growth; however, based on just the frequency of appearance in include/exclude metabolite lists (**Figure 6**), the algorithm did not have sufficient evidence to include all amino acids as biomass components even though analytical data indicates that all amino acids (in addition to other components) are present in the biomass [11]. To address this shortcoming, we treated the analytically measured biomass components as additional experimental data points for BioMog to fit. By doing this, we capture the supporting evidence that these analytical measurements provide, while still allowing BioMog to determine where the phenotypic data contradicts the inclusion of a metabolite into the biomass. *De novo* and modified biomass requirement proposals for *S. oneidensis* using this additional information are displayed in **Table 2** and **Table 3**. As can be seen, the proposals are similar to those using just growth phenotypes (**Tables 1** and **S2**); however, now all amino acids and a few additional analytically measured currency metabolites (e.g., nadh and amp) have been added. Additionally, while the list of items removed from the predefined biomass is largely the same, extracellular crosslinked peptidoglycan (peptx_e in **Figure 4B**) was retained based on the experimental measurements, even though it appeared in equal numbers of include and exclude lists indicating growth phenotypes were inconclusive regarding its essentiality.

**Table 2:** BioMog Proposed *De Novo* Biomass for *S. oneidensis* using Growth Phenotype and Experimentally Measured Biomass.

Amino Acids		Present in Predefined Biomass	No Equivalent in Predefined Biomass
L-Alanine	L-Leucine	Putrescine	O-Phospho-L-homoserine
L-Arginine	L-Lysine	nad	O-Phospho-L-serine
L-Asparagine	L-Methionine	nadh	gdptp
L-Aspartate	L-Phenylalanine	nadp	Reduced Glutathione
L-Cystine	L-Proline	nadph	2-Oxo-3-hydroxy-4-phosphobutanoate
L-Glutamine	L-Serine	dna_son	
L-Glutamate	L-Threonine	Glycogen	
Glycine	L-Tryptophan	peptx_e	
L-Histidine	L-Tyrosine	rna_son	
L-Isoleucine	L-Valine	amp	

When given additional information about biomass components measured experimentally, BioMog is able to propose more physiologically accurate components, such as including all twenty amino acids. The abbreviation peptx_e stands for extracellular cross-linked peptidoglycan, dna_son and rna_son stand for average DNA and RNA macromolecules. Other abbreviations match those in Table 1.

**Table 3:** BioMog Modified Predefined Biomass for *S. oneidensis* using Growth Phenotype and Experimentally Measured Biomass.

Added			Unmodified	Removed	
L-Alanine	L-Leucine	gdptp	amp	1,2-Diacyl-sn-glycerol 3-phosphate	Phosphatidylglycerol
L-Arginine	L-Lysine	Reduced Glutathione	dna_son	1,2-Diacylglycerol	protein_son_aerobic
L-Asparagine	L-Methionine	2-Oxo-3-hydroxy-4-phosphobutanoate	Glycogen	5-Methyltetrahydrofolate	Spermidine
L-Aspartate	L-Phenylalanine	O-Phospho-L-homoserine	nad	Acetyl-CoA	Succinyl-CoA
L-Cystine	L-Proline	O-Phospho-L-serine	nadh	acyl-glycerophosphoethanolamine	UDP-glucose
L-Glutamine	L-Serine		nadp	acyl-glycerophosphoglycerol	
L-Glutamate	L-Threonine		nadph	Coenzyme-A	
Glycine	L-Tryptophan		peptx_e	Flavin adenine dinucleotide	
L-Histidine	L-Tyrosine		Putrescine	lipopolysaccharide	
L-Isoleucine	L-Valine		rna_son	Phosphatidylethanolamine	

While BioMog is now able to capture amino acid essentiality, it will still propose the removal of certain metabolites from the predefined biomass equation based on the present model structure and results from the growth phenotype experiments. These removals may not indicate that these metabolites are non-essential but may instead indicate an experimental or network structural issue. Abbreviations match those in Table 1 and 2 with the addition of protein_son_aerobic which represents an average protein macromolecule.

### Experimental Design

In addition, to proposing and modifying biomasses, BioMog is capable of proposing experiments to interrogate the essentiality of any given metabolite (**see File S1 and File S2 for methods and implementation respectively**). Various experimental proposals involving the use of minimal media lacking essential inorganic compounds, or the deletion of genes essential to metabolite production were generated for *E. coli* predefined biomass components where BioMog indicated that little to no experimental evidence existed or the experimental evidence was conflicting (see **supplementary dataset S1**). Experiments testing for the essentiality of protoheme (pheme) and deoxythymidine triphosphate (dttp) were then conducted. The first experiment was designed to test the essentiality of pheme through the double deletion of *hemF* and *hemN*. We were unable to construct a viable double knockout mutant – implying the double knockout was lethal in LB medium – indicating this metabolite is likely essential. The second experiment, designed to test for the essentiality of dttp, was one already performed by Orth et al.[10], that is the growth phenotyping of a *ΔthyA* mutant on glucose M9 medium. However, the positive growth result contradicted findings of other publications [12], [14], [15] and led to the removal of dttp from BioMog's biomass. Since BioMog's decision to exclude dttp from the biomass was solely due to the *ΔthyA* experiments, we decided to repeat the results based on the supporting literature. Thus, growth phenotype experiments were performed evaluating the *ΔthyA* mutant's fitness in LB medium supplemented with thymine, glucose M9 minimal media, and glucose M9 media supplemented with thymine. Our results (see **Figures S1–S3 in File S1** for growth curves) suggest that *ΔthyA* is a thymine auxotroph, incapable of growth in glucose M9 media, in agreement with findings by Weiss[15]. Based on these experiments, it can be seen that the BioMog framework can be a valuable tool, not only for discovering new metabolite requirements for growth, but also for identifying potentially inaccurate experimental results within a large dataset.

## Conclusions

The BioMog framework provides a facile, fast and scalable approach to the task of generating biomass related hypotheses. Such an approach can be used to identify organism specific biomass components and improve a model's predictive power compared to using a predefined biomass equation. Alternatively, future efforts could focus on using growth phenotype data in conjunction with ^13^C and other multiomics data to further improve and refine essential biomass components. Since these approaches do not require a biomass to be defined *a priori*, it should be possible to combine metabolic flux analysis (MFA) [16] and BioMog.

Since the quality of BioMog solutions can only be as good as the experimental data used to generate them, it is important to have a large, diverse and accurate experimental dataset. This is especially true when generating biomass components *de novo*, since the method cannot include metabolites in the biomass when there is no experimental evidence to support (or refute) their inclusion as was seen with *S. oneidensis*. Presently, there are numerous methods that can be used to generate high-throughput growth and fitness data and to construct higher order genetic knockouts such as TagModule [17], TRMR [18], and MAGE [19]. Using such methods, in conjunction with the BioMog experiment generation tool, should ensure a broad evaluation of an organism's essential metabolites. Nonetheless, for organisms with robust metabolic networks, higher order knockouts may be needed to detect essential metabolites. With the *E. coli* iJO1366 network, for example, over 50% of the metabolites (after filtering out mutants resulting in large numbers of blocked metabolites) had no direct supporting or refuting evidence for their essentiality when considering experiments only involving single gene deletions. Perhaps more critically, over 40% of the components in the *E. coli* predefined biomass have no explicit evidence for their inclusion in biomass based on the single knockout datasets considered here [10]. Thus, to properly interrogate the accuracy of an organism's biomass composition it is critical that targeted higher order mutants be created.

From a mathematical perspective, BioMog works to find the best biomass requirements for the current model structure (reactions and GPR associations) and provided experimental results. As such, the proposed *de novo* and modified biomass components represent the needs of the organism assuming the model and experiments are an accurate portrayal of the organism's genetic and metabolic capabilities. If known biomass components are not proposed by BioMog then it is possible that the model or experiments are incorrect. This is most readily apparent when BioMog suggests the removal of previously reported essential metabolites such as dttp, Cu^2+^ and Ca^2+^ for *E. coli* or protein_son_aerobic for *S. oneidensis*. In these instances, it should not immediately be interpreted that these metabolites are inessential for growth (especially if these compounds have been measured experimentally to be a component of biomass) but instead this result should be considered a red flag, indicating additional experiments are needed to verify the accuracy and validity of both the model structure and experiments. In this aspect, BioMog behaves as a hypothesis generation and model interrogation tool with easy traceability (as the exclude metabolite lists allows for facile determination of experiments which conflict with metabolites being classified as essential).

Because BioMog depends on the model structure in order to make biomass proposals, it should be used in conjunction with other model refinement such as GrowMatch [3], SMILEY [6] and other knowledge gap filling algorithms for refining metabolic models when experimental results conflict with known biomass components. By using these tools in conjunction, it should be possible to add or remove reactions or GPR associations, such that a particular gene knockout and the resulting phenotype are consistent with known biomass components. For example, the biotin synthesis pathway was incomplete in the latest *S. oneidensis* genome-scale model since the enzyme for producing the precursor pimelyl-ACP was only recently discovered [20]. Consequently, it would have been impossible for BioMog to predict the essentiality of biotin; however, one of the biotin precursors S-Adenosyl-L-methionine was predicted to be an alternative biomass component. Any network structural issues that cause a metabolite to be net production blocked in the wildtype strain will prevent BioMog's ability to propose these metabolites in the biomass. There are a number of net production blocked metabolites in the wildtype (134 and 109 in *E. coli* and *S. oneidensis*, respectively) that are not biomass candidates due to an inability to synthesize precursors or due to various attached cofactors (e.g., ACP and trna) for which there is no synthesis pathway included in the model. We should note that when determining net production blocked metabolites sinks are added for all metabolites in the network, so metabolites will only be blocked if their precursors can not be produced (an inability to degrade by-products will not cause a metabolite to be net production blocked by BioMog). If some of these net production blocked metabolites are to be considered as biomass components, the model will first need to be gap filled so the metabolite of interest can be produced using methods like SMILEY and GrowMatch or by adding artificial source reactions (e.g., using reaction notation, →ACP) for precursors. Nonetheless, while various structural issues may prevent BioMog from proposing an essential end product used in biomass, it can still capture essential upstream precursors that can be produced by the network. As mentioned above, while biotin is a net production blocked metabolite in *S. oneidensis*, S-Adenosyl-L-methionine, a biotin precursor, is one of the alternative proposed biomass candidates. Thus, the process of model refinement and automated biomass proposals should be an iterative one where the model is refined, BioMog is used to modify a biomass and any unexpected results investigated and further refined using existing tools.

While valuable as a hypothesis and experimental generation tool, BioMog also has value in identifying experiments with erroneous or contradictory results. Researchers can not only use blocked metabolite lists to assess which experiments support or refute the essentiality of a particular biomass component, but also which experiments appear inconsistent either because of experimental or model inaccuracies. Moreover, BioMog biomass modification proposals can further point out potential structural flaws in an existing genome-scale model's GPR or reaction networks. For example, the recommendation removing calcium and copper from the *E. coli* biomass appears to be the direct consequence of an incomplete depiction of the transport systems for these two inorganic compounds. The iJO1366 model includes single calcium and copper transport reactions into the cytoplasm that are associated with *zupT* (b3040) and *yrbG* (b3196), respectively. The 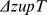
and 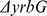 mutants are the only ones that contain Ca^2+^ or Cu^2+^ as blocked metabolites and both mutants are viable, suggesting either an incomplete picture of copper and calcium transport across the cell membrane or the non-essentiality of these metabolites. While this may have been difficult to pick out of the entire dataset, using BioMog's blocked metabolite frequencies makes finding areas of metabolism needing model improvement easier. Consequently, even if experimenters are confident in the quality of the defined biomass composition, BioMog can be a valuable component of a model curator's toolbox and supplements existing approaches [2].

Accurate knowledge of an organism's essential metabolites is critical to a model's predictive utility. Constraint-based analyses rely on a model's biomass equation to determine whether a particular genetic modification will be fatal. Failure to accurately capture this aspect of a cell can be costly both in terms of time and money when models are used in experimental design, such as strain design in metabolic engineering [21]. Such metabolite essentiality information can also be valuable for the development of new antimicrobial agents by facilitating the identification of novel drug targets using computational methods [22], [23], [24], [25]. For this application in particular, finding unique essential metabolites would be valuable for the selective and targeted treatment of specific pathogenic species. By generating more representative and organism specific biomass components, BioMog has the potential to improve the performance of algorithms that use constraint-based metabolic models.

## Methods

The biomass modification and generation (BioMog) framework allows for the identification of required biomass components from high-throughput growth and fitness experiments. The framework enables scientists to systematically make metabolic additions or removals from a predefined biomass or create new biomass equations *de novo*. Subsequently, experimentalists can use the BioMog framework to recommend additional wet lab experiments to interrogate the essentiality of particular metabolites for which data is lacking or contradictory. This process is accomplished through a series of linear, integer and mixed integer programs (LP, IP and MILP respectively), as described in detail in the following sections.

### Genome-Scale Models

BioMog was applied to genome-scale models for *Escherichia coli* (iJO1366) and *Shewanella oneidensis MR-1* (iSO783). While iJO1366 was evaluated as is, thirty-five reactions were added to iSO783 to reflect additional knowledge regarding the organism's metabolic capabilities. These modifications are summarized in **supplementary dataset S2**).

### Finding Blocked Metabolites

BioMog functions by determining which metabolites can be produced and removed from the system by the wild type strain, but not by a particular mutant. Such metabolites are referred to as blocked metabolites. The discovery of blocked metabolites was accomplished using the following MILP and is similar to that used by the GapFind algorithm [8]:
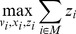
(1)

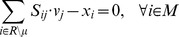
(2)

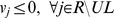
(3)

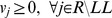
(4)

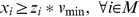
(5)

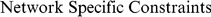
(6)


Where *z_i_* is a binary variable indicating whether a metabolite *i* can be produced, *v_j_* is a free variable for the magnitude of the flux through reaction *j*, and *x_i_* acts as a sink for metabolite *i* and is used as an indicator of metabolite production. *M* is the set of all metabolites, *R* is the set of all reactions, *μ* is a set of predefined biomass components (if any exist), *UL* is the set of reactions with non-zero upper limits and *LL* is the set of reactions with non-zero lower limits. The parameter *v_min_* sets a minimum production threshold and was set to 0.0005. Network specific constraints refer to any constraints that are particular to a given genome-scale model. For example, constraints that force reaction fluxes to zero by default, that fix P/O ratios or that set oxidase ratios to a fixed value. Blocked metabolites are those for which *z_i_* is 0 and are determined for the mutant and wild type strains used in the experiments. A set difference between the two is then performed and the remaining blocked metabolites are considered potential biomass equation candidates for inclusion or exclusion.

Note that the formulation above is written such that mass is actively removed from the system via sinks (**Eq. 2**). As a consequence, the MIP has the unique feature of finding metabolites associated with metabolic dead ends, as well as, metabolites participating in cycles that cannot be depleted from the system without violating conservation of mass. For example, in the *S. oneidensis* mutant ΔSO4249, succoa is a blocked metabolite even though flux through the metabolite is possible by running the citric acid cycle. This also means that such blocked metabolites may be implicitly essential for growth, but may be removed from the biomass equation.

### Validation of Blocked Metabolites and Generation of Blocked Metabolite Lists

Once blocked metabolites have been determined for all experiments, mutants associated with >100 blocked metabolites are filtered from consideration as they are deemed uninformative as these filtered mutants are often due to deletion of an essential early reaction (e.g., glucose transport). The set of blocked metabolites for a given mutant are given an experiment number (*e*) and organized into include (*In*), if mutant does not grow, or exclude (*Ex*), if the mutant does grow, metabolite lists. *NG* is the set of experiments where mutants did not grow and *G* is the set of experiments were mutants grew.

### Generate *De Novo* Biomass Components

Once complete include and exclude metabolite lists have been created based on the blocked metabolite lists and growth phenotyping data, the following integer program (IP) can be solved to propose, *de novo*, an optimal biomass:
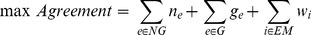
(7)

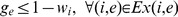
(8)

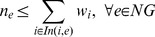
(9)

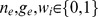
(10)


Where *n_e_* and *g_e_* are binary variables indicating agreement with no growth and growth experiments respectively, *w_i_* is a binary variable indicating whether metabolite *i* is a biomass component, *In(i,e)* and *Ex(i,e)* are mappings from a blocked metabolite to an experimental and are the include or exclude metabolite lists, respectively, and *EM* is an optional set containing metabolites that have been experimentally measured within a microbe's biomass. For most analyses EM was left as an empty set, with the exception of the *S. oneidensis* analysis using analytical biomass measurements. While not done for the purposes of this paper, it is also possible to weight individual experiments in **Eq. 7** based on one's confidence of a particular result (e.g., independent repetitions of a particular experiment). Once the maximal experimental agreement is determined, a biomass equation that best agrees with experimental results can be generated. When, multiple possible solutions exist, negative weights can be added to select more common metabolic components by preferentially including them in a new objective (**Eq. 11**) (e.g., amino acids) or positive weights can be used to find a minimal number of required biomass components (in this case all metabolites have the same positive weight).
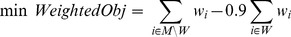
(11)

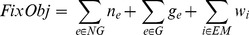
(12)

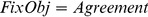
(13)


Here, *W* is the set of weighted metabolites for preferential inclusion in the biomass, *FixObj*, is the objective score obtained from running the LP using **Eq. 7–10**, and *WeightedObj* is the new weighted objective function. While not shown, **Eq. 8–10** should also be included along with **Eq. 11–13** in the weighted LP in order for the problem to be solved correctly.

### Modify Biomass Components

In addition to generating a biomass equation *de novo*, it is also possible to begin with an existing biomass equation and modify it to include likely biomass components and only remove components that fail to match experimental results. This allows one to include components for which no supporting growth phenotype data exists, but may have been demonstrated to be essential using alternative methods:
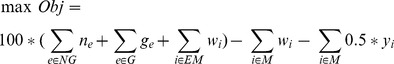
(14)

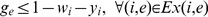
(15)

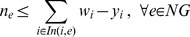
(16)

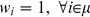
(17)

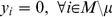
(18)

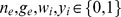
(19)


Here *y_i_* is a binary variable that removes biomass components if experiments disagree with its inclusion, *w_i_* is a binary variable that adds biomass components if experiments support their inclusion, and *μ* is the set of metabolites in the predefined biomass equation.

### Precursors and Alternative Biomass Components

For the purpose of this paper, alternative metabolites are any compound that can replace a proposed biomass compound without negatively impacting the objective function (**Eq. 7**). To determine alternative metabolites, *w_i_*, all proposed biomass components were fixed to 1 with the except for a single metabolite, *k*, for which an alternative metabolite is being sought. For this single metabolite, w_k_ was set to 0. The *de novo* minimal biomass LP is then resolved (**Eq. 8–13**) using a variant of the objective (**Eq. 11**) in which no metabolites are preferentially weighted (i.e., the set *W* is empty in **Eq. 11**). If a solution exists, then the newly added metabolite is an alternative for metabolite, *k*. The newly discovered alternative metabolite's indicator value is subsequently fixed to 0, and this process is repeated until the problem is infeasible so that all possible alternatives for that metabolite are exhausted.

Precursor metabolites are defined as upstream compounds that are essential for the production of a biomass metabolite. As such, including a particular metabolite in the biomass implicitly makes that metabolite's precursors essential. Thus, any metabolites within the predefined biomass that are found to be precursors to the *de novo* biomass components can be considered implicitly included in the *de novo* biomass for the conditions tested. Since precursor lists depend on the nutritional environment of the cell, a robust media was modeled that included all the possible conditions tested experimentally (glucose, succinate and lactate for *E. coli* and lactate, pyruvate, ammonium and nitrate for *S. oneidensis*). To determine precursors, all reaction fluxes, v_j_, were decoupled into forward and reverse directions such that *v_j_* = *v_j,for_*−*v_j,rev_* where *v_j,for_* and *v_j,rev_* are both positive variables. Then, the following formulation was used:
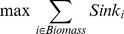
(20)

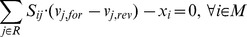
(21)

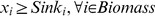
(22)

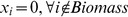
(23)

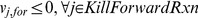
(24)

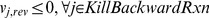
(25)

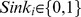
(26)


Here *Biomass* is the set of *de novo* biomass components and *Sink_i_* is a binary variable indicating if there is flux through sink x_i_. For each metabolite not contained within the *de novo* biomass, the metabolite was individually removed from the system (i.e., all associated reaction fluxes that consume the metabolite were fixed to zero), and the MIP given by **Eq. 20–26** was solved. If the cell is only viable when the metabolite is present (i.e., the cell can only produce all biomass components when the metabolite is present), then the compound was declared an essential precursor. This process was repeated using the predefined biomass to determine if any *de novo* components could be found upstream. In this LP, *KillForwardRxn* and *KillBackwardRxn* remove consuming reactions associated with the targeted metabolite in the forward and reverse directions respectively, as well, as any forward (reverse) reaction which has an upper (lower) limit equal to zero.

### Addition of Dead-End Metabolites to Biomass

Due to the addition of sinks for all metabolites when finding blocked metabolites in the BioMog framework (**Eq. 1–6**), it is possible that a biomass equation generated only from biomass components proposed using **Eq. 8–13** may result in no growth predictions for cases where the model was supposed to predict growth (i.e., *g_e_* equals 1). This occurs when a by-product of a proposed biomass component has no way of being consumed in the network for that experiment (*e*). The presence of sinks for these by-products in **Eq. 2** still allows for the biomass components to be produced when evaluating blocked metabolites. As a result these dead-end by-products need a way of being consumed in the model, which can be done by adding additional consuming reactions to the model or including them in the biomass equation as well. For example, in order to correct erroneous predictions due to dead end by-products, a few metabolites (S-adenosyl-L-homocysteine, glycolaldehyde and oxidized glutathione) were added to the iJO1366 BioMog proposed *de novo* biomass to behave as sinks for these by-products. To determine these dead end by-products, the following formulation was used:
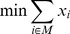
(27)

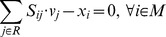
(28)

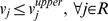
(29)

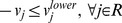
(30)

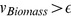
(31)

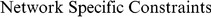
(32)


Where *v_Biomass_* is the flux through the newly proposed *de novo* biomass equations and 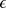 is a small number (e.g., 0.001). Network specific constraints (**Eq. 32**) covers any additional constraints specific to the network (e.g., P/O ratios in iJO1366). To avoid erroneous results due to the predefined biomass behaving as a sink, the predefined biomass equation was removed from the stoichiometric matrix for all subsequent analysis involving the BioMog biomass equation. All non-zero *x_i_*, should be considered dead end by-products of required biomass components. These should be fixed by addition of the metabolite to the biomass equation or by adding consuming reactions to the network. This process was repeated for all experiments where the model was supposed to predict growth (*g_e_* = 1). Additionally, cuts can be added to determine alternative solutions for any given experiment by fixing a proposed sink to 0.

### FBA Testing

To test the qualitative performance of the *de novo* biomass requirements, an FBA was solved as described by Feist et al. [13] for each growth and non-growth phenotype experiment for which data was collected using the predefined biomass, as well as, the *de novo* variant. Since BioMog only makes qualitative proposals, an arbitrary stoichiometric value of −0.0002 was assigned to each essential metabolite proposed. To prevent the predefined biomass from behaving as a sink, both the core and wild type biomasses were removed from the iJO1366 model when evaluating the *de novo* biomass components.

### Description of Growth Phenotype Datasets

Growth phenotyping datasets for *E. coli* and *S. oneidensis* were taken from publications by Orth et al. [10] and Deutschbauer et al. [5] respectively. For *E. coli*, the growth and no growth determinations were used as reported by in the paper for four conditions (glucose aerobic, glucose anaerobic, succinate aerobic and lactact aerobic). Additionally, all essential genes from the Keio collection [12], [26] were included as no growth mutants under glucose aerobic conditions. For *Shewanella*, a fitness score of less than −1 indicated no growth and greater than −1 indicated growth. From this dataset, we tested three conditions (lactate aerobic, pyruvate aerobic, lactate anaerobic with nitrate electron acceptor).

### Strains

All *E. coli* strains used in the experiments were derived from the Keio collection [12]. The following single knockout mutants were used: *thyA*::kan, *hemF*::kan, and *hemN*::kan as well as the parental strain, *E. coli* K-12 BW25113. Two double mutants (*ΔhemN*::kan *ΔhemF*, and *ΔhemF*::kan *ΔhemN*) were attempted to be made by first removing the *kan* gene from the single knockouts using FLP recombinase [27] and then moving the second mutation into the single knockout using P1 transduction [28]. However, no kanamycin resistant double knockouts were found. P1 phage efficacy and recipient strain sensitivity was verified using P1 transduction of the original mutation back into the kanomycin sensitive strains (i.e., by infecting P1 phage from *ΔhemN*::kan strains into kanomycin sensitive *ΔhemN* strain and *ΔhemF*::kan into *ΔhemF*) and finding robust growth on kanomycin plates following P1 transduction.

### Growth Phenotype Plate Experiments

All strains were grown in triplicate unless otherwise noted. All strains were pre-cultured for approximately 24 hours in 90% (V/V) M9 minimal medium supplemented with 2 g/L glucose and 10% (V/V) LB with 125 µg/mL thymine. Cells were washed twice with M9 minimal media containing no carbon source to remove any residual glucose and LB from precultures. Cells were then resuspended in different media, M9 minimal media with 2 g/L glucose or M9 minimal media with 2 g/L glucose and 125 µg/mL thymine, such that the starting OD_600_ measurement was ∼0.05 and cultured for at least 3 days in a Tecan plate reader at 37°C.

## Supporting Information

Dataset S1 (XLSX)

Dataset S2 (XLSX)

File S1 (DOCX)

File S2 (ZIP)
