# Supplementary material for: BioMog: A Computational Framework for the De Novo Generation or Modification of Essential Biomass Components
Source: PLoS One. 2013 Dec 5;8(12):e81322. doi: 10.1371/journal.pone.0081322 (PMC3855262; doi:10.1371/journal.pone.0081322)
Supplement: File S1 — (DOCX) [file pone.0081322.s003.docx]

| \|  \| **Page** \| \| --- \| --- \| \| **1. Supplemental Methods** \|  \| \| 1.1. Design of Experiments \| 2 \| \| **2. Supplemental Figures and Tables** \|  \| \| Figure S1. Growth curves for strains using glucose M9 media \| 3 \| \| Figure S2. Growth curves for replated strains using glucose M9 media \| 4 \| \| Figure S3. Growth curves for strains using glucose M9 media supplemented with thymine \| 4 \| \| Table S1. BioMog modifications to the iJO1366 predefined biomass using just growth phenotypes \| 5 \| \| Table S2. BioMog modifications to the iSO783 predefined biomass using just growth phenotypes \| 5 \| \| Table S3. Comparison of iJO1366 predefined biomass and that proposed by BioMog using just growth phenotypes \| 6 \| \| Table S4. Comparison of iSO783 predefined biomass and that proposed by BioMog using just growth phenotypes \| 6 \| |  |
| --- | --- | --- | --- | --- | --- | --- | --- | --- | --- | --- | --- | --- | --- | --- | --- | --- | --- | --- | --- | --- | --- | --- | --- |

**Supplemental Methods:**

**1.1 Design of Experiments**

For expected biomass components for which there were no experiments to support a particular metabolite’s inclusion or exclusion from biomass, additional experiments can be designed using the following formulation:

**Outer Objective**

|  | $\min\frac{100}{\epsilon}*\sum_{i\in bioMetab} x_{i}- delPenalty*\sum_{g\in G} (ko_{g}-1)-altPenalty* \sum_{j\in AltMedia} -f_{j}- essentialPenalty*\sum_{j\in Essential} {(m}_{j}-1)$ | (1) |
| --- | --- | --- |

**Inner Problem**

|  | $\max_{v_{j},x_{i}} \sum_{i\in bioMetab} x_{i}$ | (2) |
| --- | --- | --- |
|  | $\sum_{j\in R\backslash Blocked} S_{ij}*v_{j}=0, \forall i\in M\backslash biometab$ | (3) |
|  | $\sum_{j\in R\backslash Blocked} S_{ij}*v_{j}-x_{i}=0, \forall i\in biometab$ | (4) |
|  | $v_{j}\leq v_{j}^{upper}, \forall j\in R\backslash Blocked$ | (5) |
|  | ${-v}_{j}\leq v_{j}^{lower}, \forall j\in R\backslash Blocked$ | (6) |
|  | $v_{j}=0, \forall j\in DelSet_{j}, if a_{j}=0$ | (7) |
|  | $-v_{j}\leq0,$ $\forall j\in MediaSet, if h_{j}=0$ | (8) |
|  | $-v_{j}\leq0, \forall j\in Exch\backslash MediaSet$ | (9) |
|  | $-\mu^{''}\leq-\epsilon$ | (10) |
|  | Network Specific Constraints | (11) |

**Outer Constraints**

|  | Deletion Rules | (12) |
| --- | --- | --- |
|  | Media Rules | (13) |

The outer objective function (**Eq. 1**) attempts to find a solution where the metabolite of interest, *biometab*, is production blocked while minimizing the number of deletions, *ko­_g_*, and media alterations – either auxotrophic supplements, f_j_, or removal of essential (typically inorganic) metabolites, m_j_. For the purposes of this project, the following penalty variables were used: *epsilon* was set to 0.01, *delPenalty* was set to 1, *altPenalty* was set to 0.25 and *essentialPenalty* was set to 1.

The inner problem is designed to maximize the flux of the metabolite of interest through the sink, *x_i_*_,_ to ensure that in any valid optimal proposal the blocked metabolite will be production and not consumption blocked. **Eq. 3** and **4** are material balances without and with an association to the metabolite of interest. **Eq. 5** and **6** are standard flux constraints while **Eq. 7 and 8** are conditional constraints associated with reaction deletions, *a­_j_*, and media selection, *h­_j_*. **Eq. 10** ensures that the proposed mutant is viable (i.e., there is flux through *µ’’*, the modified biomass flux). Here it is assumed that the metabolite of interest is not an essential metabolite; thus, if the originally proposed biomass includes this metabolite, the stoichiometric matrix biomass entry is modified such that the metabolite of interest is no longer a member.

Outer constraint deletion and media rules are as defined by Tervo and Reed [10] with the addition of another media subset, *essential*, used to handle the removal of essential inorganic metabolites. The dual problem and the complete media and deletion rules are provided as a **supplementary file S2** for those interested.

**Supplemental Figures and Tables:**


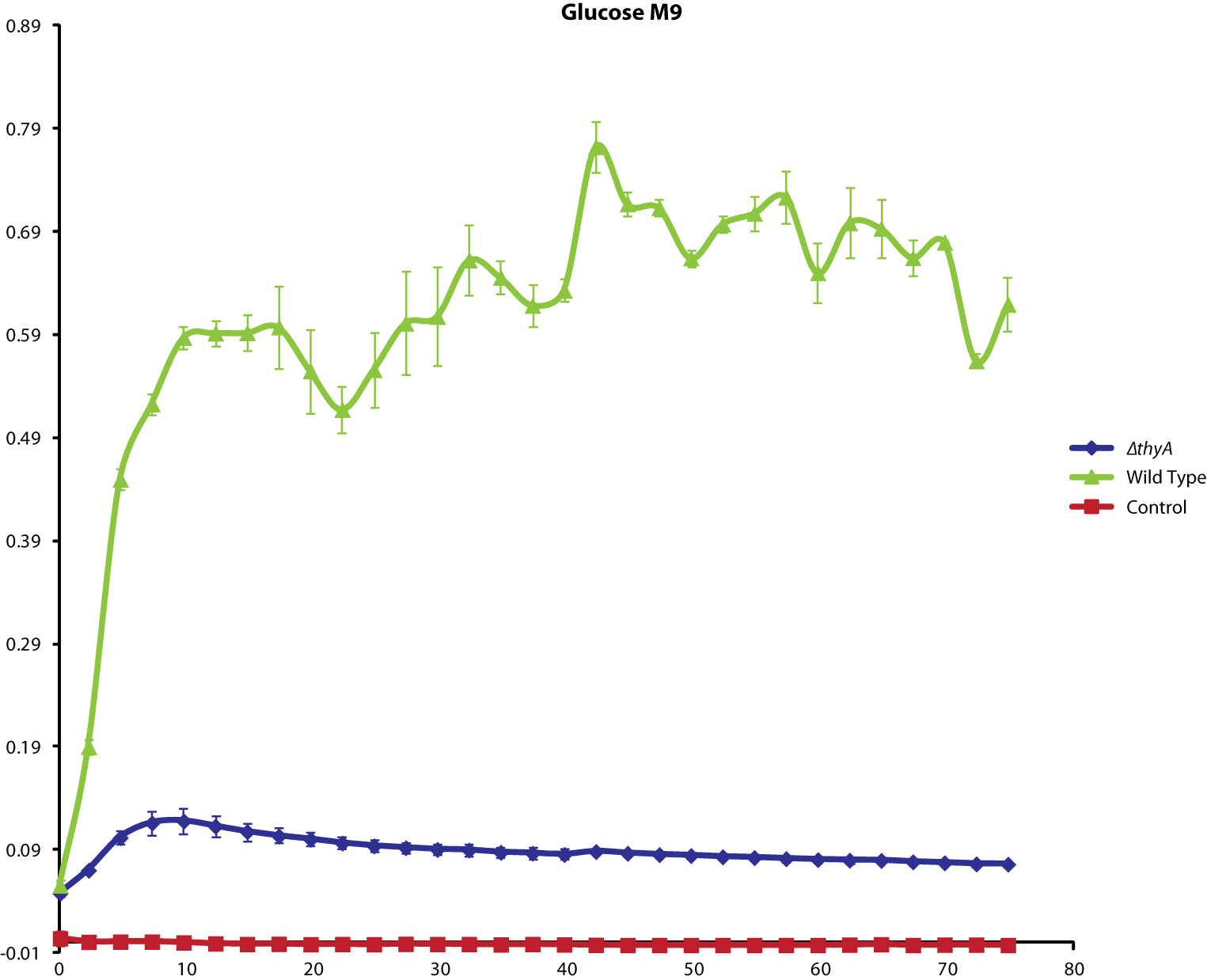


Figure S1: Growth curves for strains using glucose M9 media. While the wild type strain exhibited healthy growth, the Δ*thyA* mutant was fatal. The very slight growth is likely attributable to residual LB + thymine from the strains preculture (see Figure S2). In this and the remaining growth experiments, strains were grown in triplicate, and the error bars represent the standard deviation of those experiments.


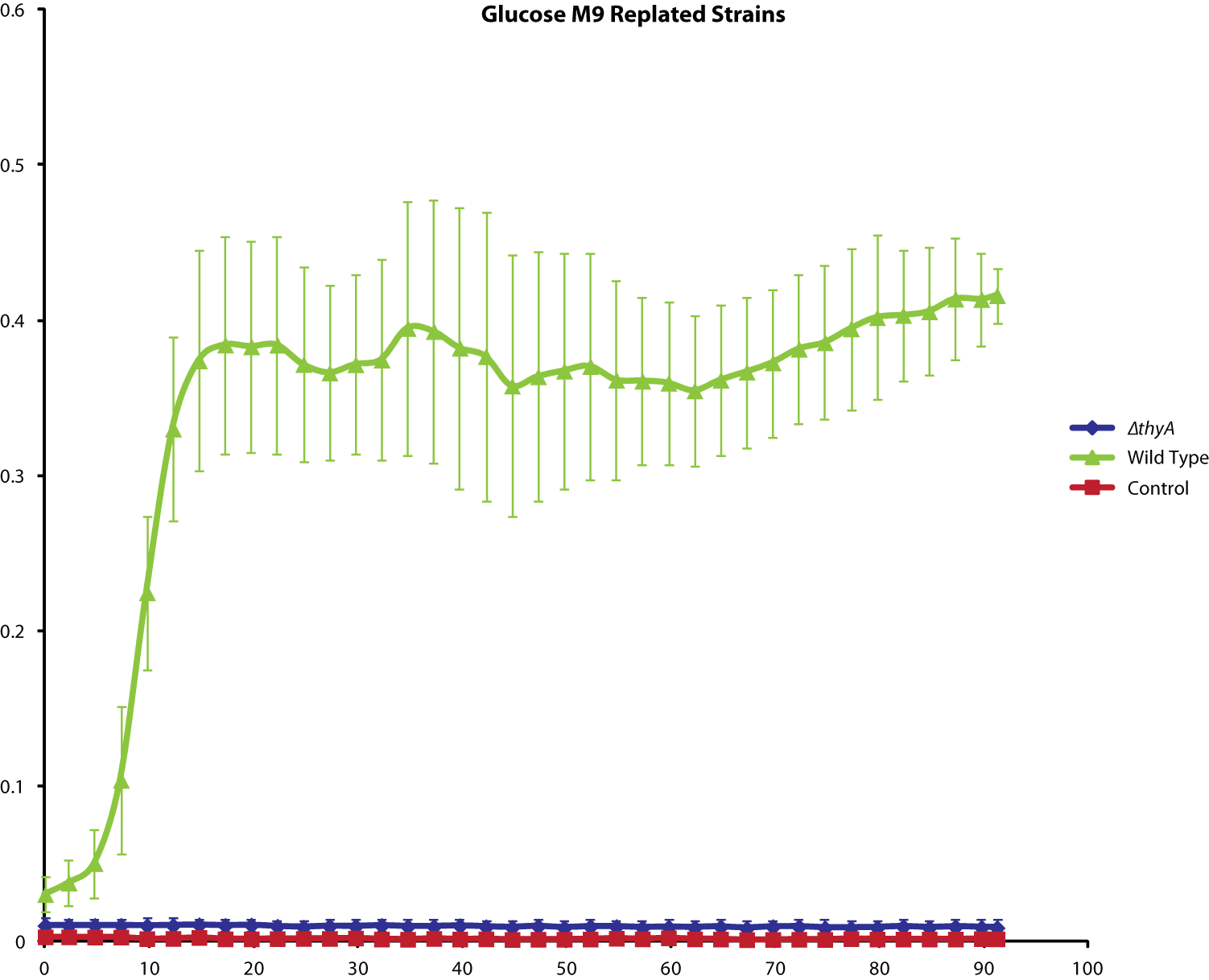


Figure S2: Growth curves for transferred strains using Glucose M9 media. To verify that the Δ*thyA* was unable to grow in unsupplemented glucose M9 medium, a 10 µL aliquot was transferred from each cell from the original 96 well plate experiments in Figure S1 into fresh media. No subsequent growth was observed for the Δ*thyA* mutant.


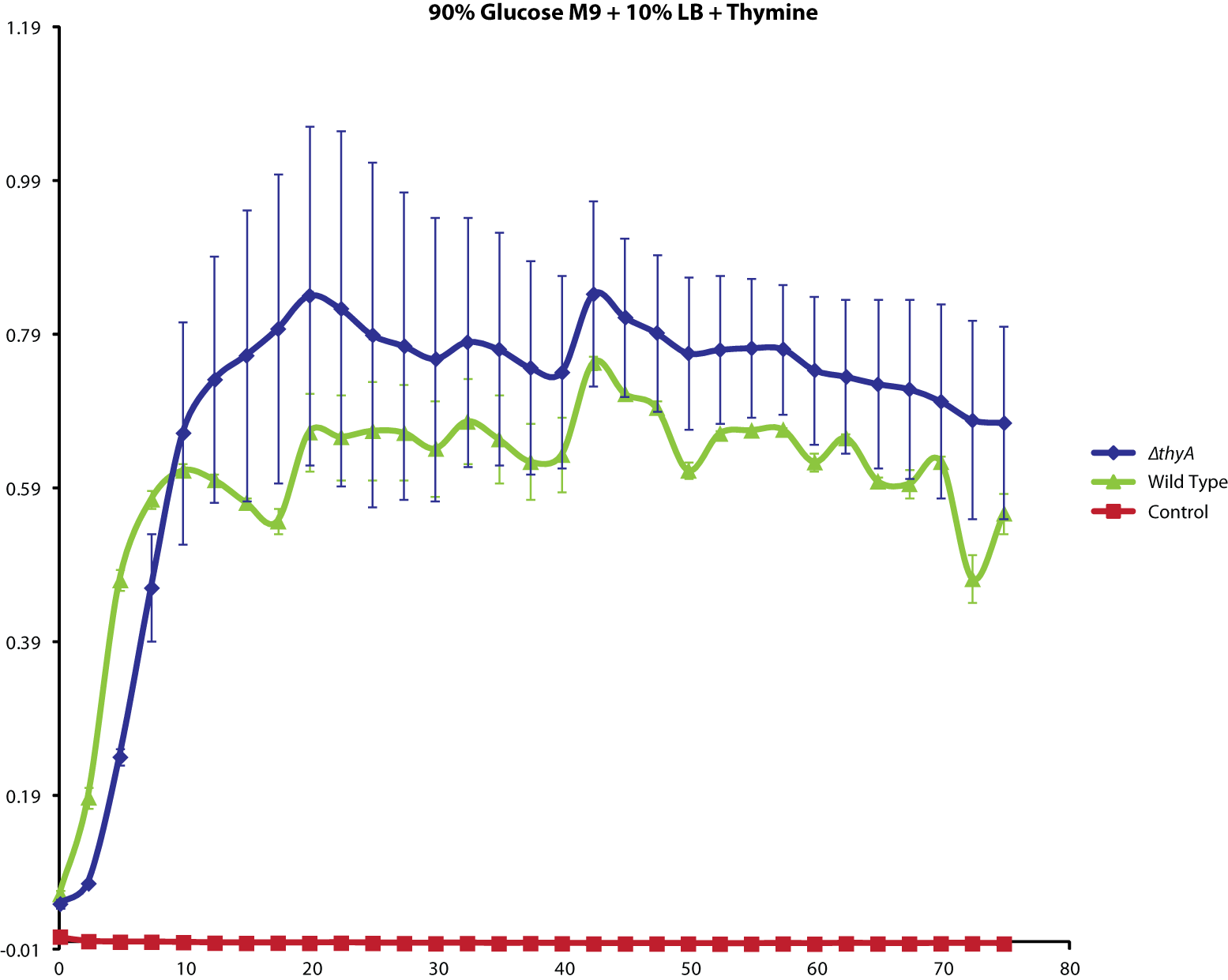


Figure S3: Growth curves for strains on glucose M9 media supplemented with thymine. By growing Δ*thyA* on media that also contains thymine, we see a full recovery of the Δ*thyA* strain’s growth suggesting that Δ*thyA* is a thymine auxotroph.

Table S1: BioMog modifications to the iJO1366 predefined biomass using just growth phenotypes. Added metabolites are compounds that BioMog recommends to be added to the core biomass to account for unexplained no growth phenotypes. Unchanged metabolites are those biomass components for which there was no or insufficient evidence for removal, while the removed category is composed of biomass components whose removal would improve the models growth phenotype predictions. No analytically measured metabolites were used in the BioMog objective (i.e. EM was an empty set).

| **Modified Biomass – *E. coli*** | | | | | | | |
| --- | --- | --- | --- | --- | --- | --- | --- |
| **Added** | **Unchanged** | | | | | | **Removed** |
| 23dhbzs | UDCPDP | bmocogdp | fe2 | leu-L | ni2 | sheme | ca2 |
| 2omhmbl | 10fthf | btn | fe3 | lys-L | pe160 | so4 | cu2 |
| 3php | 2fe2s | cl | gln-L | met-L | pe160_p | thf | dttp |
| 5fthf | 2ohph | coa | glu-L | mg2 | pe161 | thmpp |  |
| glu5p | 4fe4s | cobalt2 | gly | mlthf | pe161_p | thr-L |  |
| pgp180 | ala-L | ctp | gtp | mn2 | phe-L | trp-L |  |
| seln | amet | cys-L | h2o | mobd | pheme | tyr-L |  |
|  | arg-L | datp | his-L | murein5px4p_p | pro-L | utp |  |
|  | asn-L | dctp | ile-L | nad | pydx5p | val-L |  |
|  | asp-L | dgtp | k | nadp | ribflv | zn2 |  |
|  | atp | fad | kdo2lipid4_e | nh4 | ser-L |  |  |

Table S2: BioMog modifications to the *Shewanella* predefined biomass using just growth phenotypes. Added metabolites are compounds that BioMog recommends to be added to the core biomass to account for unexplained no growth phenotypes. Unchanged metabolites are those biomass components for which there was no or insufficient evidence for removal, while the removed category is composed of biomass components whose removal would improve the models growth phenotype predictions. No analytically measured metabolites were used in the BioMog objective (i.e. EM was an empty set).

| **Modified Biomass – *S. oneidensis*** | | | | |
| --- | --- | --- | --- | --- |
| **Added** | | **Unchanged** | **Removed** | |
| 1pyr5c | phthr | amp | 12dag3p | peptx_e |
| 34hpp | pser-l | dna_son | 12dgr | pgly |
| 4mop | trp-l | glycogen | 5mthf | protein_son_aerobic |
| amet |  | nad | accoa | spmd |
| arg-l |  | nadh | agpe | succoa |
| cinnm |  | nadp | agpg | udpg |
| gdptp |  | nadph | coa |  |
| glucys |  | ptrc | fad |  |
| his-l |  | rna_son | lps_so |  |
| phom |  |  | pe |  |

Table S3: Comparison between *E. coli* predefined and BioMog proposed biomass components using just growth phenotypes. Overlap indicates metabolites that are present in the predefined biomass and are components (or their alternatives) of the *de novo* biomass components. Essential precursors are metabolites whose removal from the network under the tested conditions would result in a no growth phenotype (i.e., their essentiality is implied by the proposed components of the BioMog biomass). Abbreviations match those used in the iJO1366 model. No analytically measured metabolites were used in the BioMog objective (i.e. EM was an empty set).

| *E. coli* Biomass Comparisons | | | | | | | | |
| --- | --- | --- | --- | --- | --- | --- | --- | --- |
| **De novo Biomass** | **Overlap** | | | **iJO1366 –**  **Essential Precursors** | | | **iJO1366 - Uniques** | |
| 23dhbzs | ctp | murein5px4p_p | thmpp | 10fthf | gly | utp | 4fe4s | k |
| 2omhmbl | dctp | nadp | trp-L | 2fe2s | gtp |  | asn-L | mg2 |
| 5fthf | arg-L | pe160 | UDCPDP | 2ohph | h2o |  | bmocogdp | mlthf |
| clpn181_p | btn | pe160_p |  | ala-L | met-L |  | ca2 | mn2 |
| glu5p | coa | pe161 |  | amet | nad |  | cl | mobd |
| pser-L | fad | pe161_p |  | asp-L | nh4 |  | cobalt2 | ni2 |
| Slnt | his-L | phe-L |  | atp | ribflv |  | cu2 | val-L |
|  | ile-L | pheme |  | cys-L | ser-L |  | datp | zn2 |
|  | kdo2lipid4_e | pro-L |  | fe2 | thf |  | dgtp |  |
|  | leu-L | pydx5p |  | gln-L | thr-L |  | dttp |  |
|  | lys-L | sheme |  | glu-L | tyr-L |  | fe3 |  |

Table S4: Comparison between *S. oneidensis* predefined and Biomog proposed biomass components using just growth phenotypes. Categories are identical to those in table 2 with the exception of De Novo – Essential Precursors which is composed of metabolites from the de novo biomass that are precursors to the predefined biomass (specifically, upstream metabolites of protein_son_aerobic in this instance). No analytically measured metabolites were used in the BioMog objective (i.e. EM was an empty set).

| ***S. oneidensis* Biomass Comparisons** | | | | | |
| --- | --- | --- | --- | --- | --- |
| **De Novo Biomass** | **Overlap** | **iSO783 –**  **Essential Precursors** | **De Novo –**  **Essential Precursors** | **iSO783- Uniques** | |
| gthrd | dna_son | 5mthf | arg-l | 12dag3p | pgly |
| gdptp | rna_son | accoa | his-l | 12dgr | protein_son_aerobic |
| ohpb |  | amp | leu-l | agpe | ptrc |
| phom |  | coa | met-l | agpg | spmd |
| pser-l |  | nad | phe-l | fad | udpg |
|  |  | nadh | pro-l | glycogen |  |
|  |  | nadp | trp-l | lps_so |  |
|  |  | nadph | tyr-l | pe |  |
|  |  | succoa |  | peptx_e |  |
